# An Extended Correlation Dimension of Complex Networks

**DOI:** 10.3390/e23060710

**Published:** 2021-06-03

**Authors:** Sheng Zhang, Wenxiang Lan, Weikai Dai, Feng Wu, Caisen Chen

**Affiliations:** 1 School of Information Engineering, Nanchang Hangkong University, 696 Fenghe South Avenue, Nanchang 330063, China; falwwk@163.com (W.D.); 1804085211016@stu.nchu.edu.cn (F.W.); 2 Military Exercise and Training Center, Academy of Army Armored Force, Beijing 100072, China; caisenchen@163.com

**Keywords:** fractal property, correlation dimension, weighted networks, small-world network

## Abstract

Fractal and self-similarity are important characteristics of complex networks. The correlation dimension is one of the measures implemented to characterize the fractal nature of unweighted structures, but it has not been extended to weighted networks. In this paper, the correlation dimension is extended to the weighted networks. The proposed method uses edge-weights accumulation to obtain scale distances. It can be used not only for weighted networks but also for unweighted networks. We selected six weighted networks, including two synthetic fractal networks and four real-world networks, to validate it. The results show that the proposed method was effective for the fractal scaling analysis of weighted complex networks. Meanwhile, this method was used to analyze the fractal properties of the Newman–Watts (NW) unweighted small-world networks. Compared with other fractal dimensions, the correlation dimension is more suitable for the quantitative analysis of small-world effects.

## 1. Introduction

Recently, revealing and characterizing complex systems from a complex networks perspective has attracted attention. Network theory can fundamentally reshape the approach to the complexity of systems and solve various problems [1]. The studies of intricate topology contribute to understanding and characterizing the complexity of the network [2]. This macroscopic property of complex networks has been the focus of intense scientific activity [3]. Specifically, the small-world property [4] and the scale-free [5] property were separately found in many networks. These findings accelerate the study of the impact of network structure on various dynamical processes. In a small-world network, most nodes can be reached from each other node by a small number of hops or steps. Many empirical networks show a small-world effect, helping to study social networks, biological neural networks, and epidemiological processes [6,7,8]. In addition, the network’s dimension can be used to analyze the dynamics of networks. Furthermore, it is also one of the most fundamental quantities to analyze systems topology and physical properties.

Dimensions are described as topological measurements of their coverage characteristics [9]. The dimension of the mathematical space is informally considered as the minimum number of coordinates and is typically an integer, but the fractal dimension is not necessarily an integer. Mandelbrot proposed the concept of fractal in geometry [10], and the fractal dimension is widely used in many fields to describe the fractal pattern of the systems. Song et al. extended the fractal dimension to complex networks and found that many real-world networks have self-repetitive structures at all scales [11,12]. The network dimension is one of the key concepts to understand network topology and network dynamic process [13]. The fractal dimension can be used to quantitatively analyze the self-similarity or fractal property of networks. In recent years, there have been many studies on the fractal dimension of complex networks, and researchers have studied the fractal properties of networks from different perspectives. Wen et al. proposed the information dimension [14,15,16] of weighted complex networks based on the box covering algorithm (BCANw) of weighted complex networks. Huang et al. considered the node degree information and the edge weight information connected to the node, from the perspective of strength volume [17] examine the fractal characteristics of the weighted network. Sometimes, a single fractal exponent is not enough to characterize the fractal properties of the system. Multifractal analysis can provide a continuous spectrum of dimension exponents for describing the fractal patterns [18]. Song et al. proposed a modified sandbox algorithm to study the multifractal problem of weighted networks [19]. In complex networks, the fractal analysis is a useful tool that has been proven in many fields such as nonlinear time series [20], economic systems [21,22], and physical phenomena [23]. For example, Li et al. studied the vulnerability of the network from two perspectives: the connection mode between hub nodes and the fractal dimension of the network [24].

In complex networks, there are many studies on fractal dimension. Li et al. redefined the pheromone-updating rules and heuristic rules, proposed a heuristic algorithm, named the max–min ant colony algorithm, which can reduce the number of boxes [25]. Zhao et al. proposed a fractal dimension estimation method for RGB color images [26]. In addition, there are many kinds of research on the correlation dimension, but each of them has its limitations. Lacasa et al. proposed a method for correlation dimension in complex networks, and it is only applicable to the networks in coordinate space [27,28]. Rosenberg defined the correlation dimension in the finite unweighted and undirected rectilinear gird [29,30]. Wang et al. [31] studied the correlation dimension in the planar networks. The value of the correlation dimension depends on the distance and the number of node pairs. The small-world effect is that the average distance between any two nodes in the network increases logarithmically with the increase in the total number of network nodes. The correlation dimension and the small-world effect are related to the distance of node pairs, so there may exist an association between them. In this paper, we applied the correlation dimension to the small-world network to quantitatively analyze the small-world effect. In addition, the current method of calculating the correlation dimension cannot be used for weighted networks.

The edge-weights of complex networks exhibit the strength of the correlation among its components and are coupled into a topology for more accurately representing the network structure. For instance, edge-weights in the scientists’ cooperative network can represent the strength of cooperation. Moreover, in the aviation network, they can represent the flight traffic of the two places. The calculation of the rich-club effect in the real-world-weighted networks is completely different from unweighted representations [2]. Weighted quantities have a specific correlation with potential network topology [3]. However, the box-covering method proposed by Song et al. for calculating the fractal dimension [32] cannot be applied to the weighted networks. Similarly, previous methods for calculating correlation dimensions cannot be adapted to weighted networks. Wei et al. chose the box size by accumulating the sorted edge-weights and extended the box-covering method to the weighted networks [33]. Wei’s method was denoted by BCANw and proven to be valid for calculating the information dimensions [14,34] and volume dimensions [17] in weighted complex networks. Inspired by the BCANw algorithm, this paper comprehensively defines the selection formula of the size *r* of the unweighted network and the weighted network and extends the correlation dimension to the weighted network, which is closer to the fractal theory dimension than other weighted network dimensions in most cases. The correlation dimension can be used to distinguish between chaotic and truly random behavior in chaotic systems [35]. Furthermore, researchers have tried to study chaotic sequences from the perspective of weighted complex networks [36]. Therefore, the correlation dimension should be extended to adapt to the real-world weighted networks and contribute to the studies of chaotic signals from the perspective of complex networks.

## 2. Preliminaries

### 2.1. Newman–Watts Small-World Networks

The Newman–Watts model [37] is a random graph generation model for producing graphs with small-world properties and denoted by NW networks. Let *G* be an undirected graph with *N* nodes and each node has *K* (assumed to be an even integer for symmetry) neighbors, and the construction of the network starts with the nearest neighbors regular ring lattice. Shortcuts are added with the probability *P* between unconnected nodes. The probability *P* reflects the shortcuts density of the network. The typical distance *L* between two chosen nodes scales as the logarithm of the number of nodes *N*, i.e., L∝logN. There have been many studies on the fractal characteristics of small-world networks [38,39,40]. Rozenfeld et al. used renormalization group (RG) theory to explain the coexistence of the seemingly contradictory fractal and small-world phases [38]. This means that we can study the small-world network through the fractal of complex networks, so as to reveal the relationship between the correlation dimension and the small-world phenomena. Moreover, the correlation dimension is closely related to the distance of the node pairs, which means that it has a specific relationship with the small-world property. Song et al. pointed out that the NW small-world network has self-similar properties, and box-covering methods [11,32] are usually used to quantify the fractal dimensions of networks. Since finding the optimal coverings in Song’s method is an NP-hard problem, estimating the fractal dimension by correlation dimension can the avoid NP problem, and it is an effective alternative method [27]. We applied the correlation dimension method to NW small-world networks. The fractal properties and the factors affecting the correlation dimension are studied.

### 2.2. Correlation Dimension of Complex Networks

The correlation dimension was originally introduced by the Grassberger–Procaccia to measure the strange attractor in chaos theory [35,41,42]. Relying on an extension of the Grassberger–Procaccia algorithm, Lacasa et al. proposed a method for the correlation dimension of complex networks embedded in *m*-dimensional space [27]. Lacasa’s approach requires that it be embedded in *m*-dimensional space by a random walker navigating the network, and only applies to a network with the geometric coordinates of each node. Rosenberg defines the correlation dimension in the finite unweighted and undirect rectilinear grid [29]. Wang et al. proposed a method for calculating the correlation dimension of an unweighted network [31]. Wang’s method is implemented as follows. Let *G* be an unweighted network with *N* nodes and *E* edges. The correlation sum function C(r) is defined as the fraction of node pairs whose distance is less than *r*:

(1) $$C\left(r\right)=\frac{2\sum_{i<j}\theta \left(r-d_{ij}\right)}{N(N-1)},$$

where $d_{ij}$ represents the distance between node *i* and *j*. θ(x) is the Heaviside step function, when x≥0, θ(x)=1, and when x<0, θ(x)=0. Rosenberg in [43] pointed out that if the network has fractal property, then C(r) will scale with distance *r* as

(2) $$C\left(r\right)∼r^{\beta },$$

where exponent β is the correlation dimension of the network. The scaling distance *r* in the unweighted network increases from integer 1 to the diameter of the network. The correlation sum function C(r) should be calculated for each scaling distance *r*. If there exists a scaling region on the log–log plot of C(r) as a function of *r*, then a straight line can be fitted by a least-squares method in that region. The slope of that fitting line is the value of the correlation dimension. If numerous non-integer edge-weights exist in the network, the size *r* cannot be simply integer-incremented like in an unweighted network. The above method for calculating the correlation dimension will be subject to restrictions.

### 2.3. The Distance between Nodes

The value of edge-weights in the weighted network can be determined according to different needs to express different physical meanings and the strength of correlation between nodes. Coupling edge-weights to the network can describe its topological characteristics more accurately. In the weighted network, the shortest path between node *i* and node *j* is denoted as $d_{ij}$, and is defined as

(3) $$d_{ij}=min\left\{w_{ij_{1}}+w_{j_{1}j_{2}}+\cdots +w_{j_{m-1}j_{m}}+w_{j_{m}j}\right\}$$

where $w_{ij}$ is the weight value of the edge of the directly connected node *i* and *j*, $j_{m}\left(m=1,\;2,\;\cdots \right)$ are the IDs of nodes. The minimum function is the minimum of all possible combined paths from node *i* to node *j*, that is, the shortest path length. It is obvious that the shortest path defined by the above equation will increase with the increase in the weight value, as is the case with the actual traffic network [3]. However, there is also a weighted network, such as the scientist cooperative network [44], in which the more scientists cooperate, the greater the weight, but the shorter the distance between them, so another definition of the shortest path is given:

(4) $$d_{ij}=min\left\{w_{ij_{1}}^{-1}+w_{j_{1}j_{2}}^{-1}+\cdots +w_{j_{m}-1j_{m}}^{-1}+w_{j_{m}j}^{-1}\right\}$$

When analyzing the weighted network, defining the shortest path depends on the specific meaning expressed by the weight value, and then analyzed the specific problems in detail. If the weight type is dissimilarity weight, then Equation (3) is used, and the distance is proportional to the weight. If the weight type is similarity weight, then Equation (4) is used because the weight is inversely proportional to the distance. A common method for solving the shortest path to a network is Dijkstra’s algorithm [45].

## 3. The Extended Method of Correlation Dimension

In a weighted network, sometimes the edge-weights are not an integer. If the value of size *r* is still in integer increment, it causes correlation sum loss on the non-integer scale. Estimates of correlation dimension will be affected. Inspired by the BCANw algorithm, the size *r* is determined by the edge-weights. For a given weighted network, all the edge-weights are sorted from small to large after removing duplicates, and the sorting set $W=[w_{1},\;w_{2},\;\cdots ,\;w_{M-1},\;w_{M}]$ is obtained. The following formula is used to obtain the values of *r*:

(5) $$r_{k}=\left\{\begin{array}{cc} \sum_{i=1}^{k}w_{i},\;\left(k\leq M\right) & \\ \left(k-M\right)w_{M}+\sum_{i=1}^{M}w_{i},\;\left(k>M\right)\;, \end{array}\right.$$

where *k* is the ID of the radius *r*, and *M* is the number of the edge-weights after removing duplicate values. For weighted networks, the selection of scaling distance *r* is obtained by the first part of Equation (5) to achieve the effect of obtaining *r* through the accumulation of edge-weights; for unweighted networks, and all edge-weights are the same and are generally recorded as 1, then there is only one element in the set *W*, and the selection of the scaling distance *r* is obtained through the second part of Equation (5), to achieve the effect that *r* increases one by one. This ensures that the final size *r* is not smaller than the diameter of the network, and the algorithm is also applicable to the unweighted network when considering k>M. The algorithm steps are as follows:

1. Firstly, all the edge-weights are sorted from small to large after removing duplicates as $\left(w_{1},\;w_{2},\;\cdots ,\;w_{M-1},\;w_{M}\right)$. Set the initial size $r=w_{1}$;
2. For a given size *r*, the correlation sum C(r) is calculated by Equation (1), where the $d_{ij}$ is obtained by Equation (3) or Equation (4) according to different network edge-weight types. If it is the dissimilarity weight, Equation (3) is used, otherwise Equation (4) is used;
3. The next size *r* is accumulated by Equation (5);
4. Repeat step 2 and step 3 until *r* is not less than the diameter of the network;
5. Use least-squares method to fit C(r) as function of *r* in the scaling region on the log–log plot. The slope of fitting line is the correlation dimension $d_{c}$.

## 4. Correlation Dimension of Newman-Watts Small-World Network

**Figure 1:**
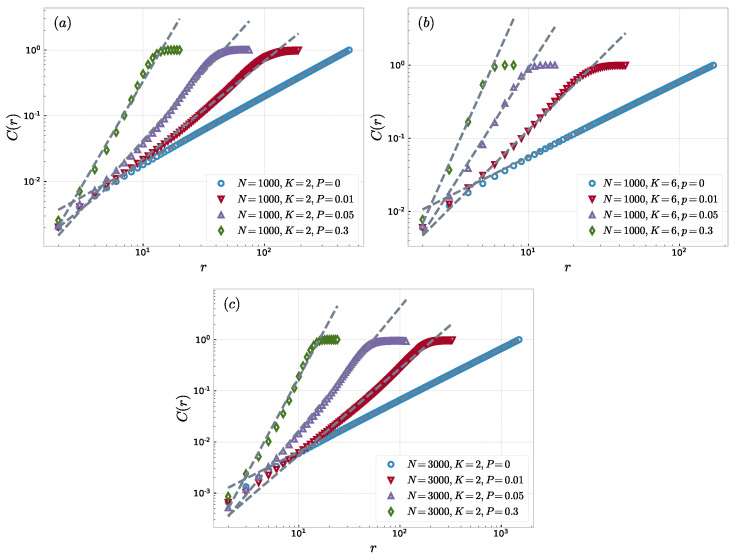
The correlation sum function C(r) within the size *r* in a NW small-world networks with various probability *P*, where the dashed lines are the experimental fitting lines: (**a**) N=1000, K=2; (**b**) N=1000; K=6; and (**c**) N=3000, K=2.

In this section, the method is used to study the fractal property of the NW small-world network. The results are shown in Figure 1. We found that in a small-world network with a different probability *P*, *r* can scale with the correlation sum C(r) size, and the correlation dimension increases significantly with the increase in probability *P*. We found that fractal properties were found in NW small-world networks with different parameters. When the probability of first-order phase transition P=0 [37], the correlation dimension $d_{c}=0.99$ has no tail distribution. Although the value of *K* or *N* is large, the correlation dimension tends towards 1 during this phase. A short tail appears when P=0.01. As *P* increases, numerous shortcuts will be added to the network and the average distance of the nodes will decrease. Therefore, the correlation sum will also increase. We also found that the initial number of neighbors in the NW small-world network is an important factor in the small-world network. When the number of network nodes *N* and the probability *P* are constant, the value of the correlation dimension of the network increases with the increase in number of neighbor nodes. Some numerical results are shown in Table 1. However, Guo et al. found that the fractal dimension $d_{V}$ based on the volume of a node is independent of *K* [46]. This means that volume dimensions do not fully reflect the nature of the small world. For another volume dimension $d_{VW}$, proposed by Wei et al., is based on the degree of nodes [47]. This method allows *K* to be quantitatively reflected in the fractal dimension $d_{VW}$ while making $d_{VW}$ independent of the number of nodes *N*. The added shortcuts have different effects on different sizes of networks. The correlation dimension can reflect the impact of *K* and *P* on networks of different sizes. This makes the correlation dimension become the appropriate index that quantifies the network small-world effect.

## 5. Correlation Dimension and Fractal Properties of Weighted Networks

### 5.1. Correlation Dimension of Synthetic Weighted Fractal Networks

**Figure 2:**
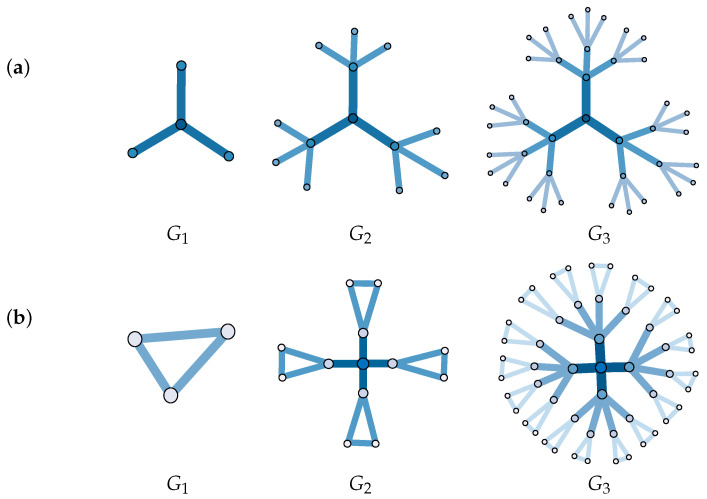
$G_{1}$ is the first generation of the networks. The different colors of the edges represent different weights: the lighter the color, the smaller the weight: (**a**) “Sierpinski” WFN, s=3; (**b**) “Cantor dust” WFN, s=4.

Our method was applied to weighted networks. To validate the method, we first applied the algorithm to the weighted synthetic fractal networks, which are the “Sierpinski” network and “Cantor Dust” triangle network [48]. The weighted fractal network (WFN) contains small copies of the entire network in distorted and degenerate forms [10]. The WFNs were constructed by *iterated function systems* [49,50]. These two WFNs are controlled by two parameters, the number of copies s>1 and the scaling factor 0<f<1. The construction processes of WFNs are shown in Figure 2. The fractal dimension of the network is also a self-similar dimension, and its theoretical calculation is as follows:

(6) $$d_{fract}=-\log s/\log\left(f\right)$$

We applied the methods to the WFNs with various scaling factor *f*. Due to the limitation of computing capability, we iterated the “Sierpinski” WFN to the eighth generation $G_{8}$. The WFN $G_{8}$ has 9841 nodes and 9837 edges. The set of non-repetitive edge-weights is $W=[f^{n-1},f^{n-2},\cdots ,f^{1},1]$. If f=1/2, the edge-weights $W=[\frac{1}{2^{7}},\frac{1}{2^{6}},\cdots ,\frac{1}{2},1]$. The minimum edge-weight is the value of initial $r_{1}$, i.e., $r_{1}=1/2^{7}$. The correlation sum $C(r_{1})$ is calculated by Equation (1). The edge-weights of WFNs do not represent any actual physical meaning. We use Equation (3) to obtain the shortest distance between nodes. The next size $r_{2}=1/2^{7}+1/2^{6}$, and the following scaling size will continue to accumulate until the *k*th scaling size $r_{k}$ is not less than the diameter of the network. If there exists a scaling region on the log–log plot of C(r) as a function of *r*, the straight line in that region is fitted by the least-squares method. The slope of the fitting line is the correlation dimension $d_{c}$.

**Figure 3:**
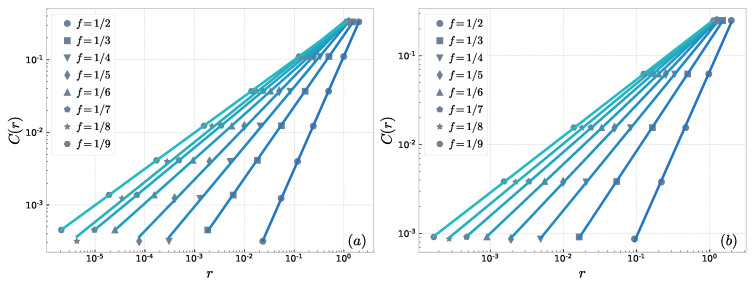
The fractal analysis of weighted fractal network with various scaling factors *f* by using correlation dimension: (**a**) “Sierpinski” WFN, s=3; and (**b**) “Cantor Dust” WFN s=4.

**Figure 4:**
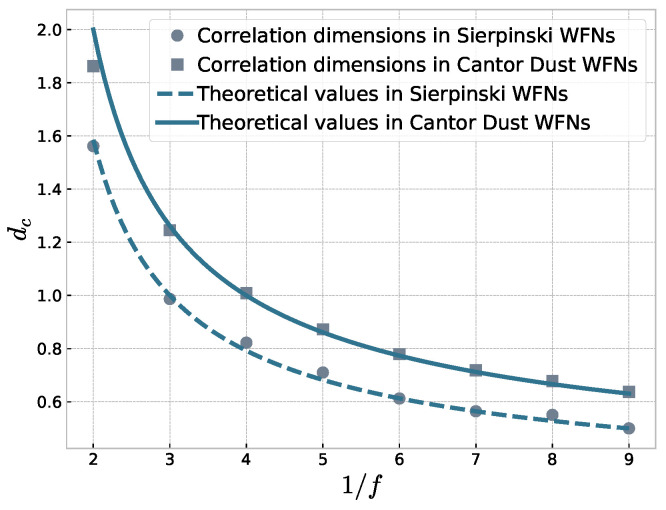
The curves are the theoretical calculation. The scatters represent the correlation dimension of two WFNs with different scaling factors *f*.

The log–log plots of *r* and C(r) with various scaling factors *f* in “Sierpinski” WFN are shown in the part (a) of Figure 3. The result shows that the “Sierpinsk” WFN has a strongly fractal property at different scale factors. Similarly, we iterate the “Cantor Dust” WFN to the sixth generation $G_{6}$ and $G_{6}$ has 13,653 nodes and 17,748 edges. The calculation results are shown in part (b) of Figure 3. In “Sierpinski” WFN, the number of copies s=3, thus its theoretical fractal dimension is $d_{fract}=-\log\left(3\right)/\log\left(f\right)$. The theoretical fractal dimension of “Cantor Dust" WFN is $d_{fract}=-\log\left(4\right)/\log\left(f\right)$. We compare the theoretical computation with the computation of correlation dimensions shown in Figure 4. The correlation dimension is very close to the theoretical fractal dimension in either the“Sierpinski” WFN or the “Cantor Dust” WFN, so our method is effective to quantitatively study the fractal properties of the weighted fractal network. We calculated some fractal dimensions, including the correlation dimensions, information dimensions, and the dimensions calculated by BCANw method. These three dimensions and the theoretical dimensions with different scales are shown in Table 2 and Table 3. The results show that compared with other fractal dimensions, the correlation dimension of WFNs is close to the theoretical value.

### 5.2. Fractal Properties of Real-World Weighted Complex Networks

We applied the method to study the fractal properties of four real-world weighted networks. Netscience is a collaborative network of the co-author in network science [44], Cgscience is a collaborative network in computational geometry, USAir is a US Airlines weighted network [51], and Coplant is a biological network that captures global cellular connectivity within the hypocotyl of plants [52].

**Figure 5:**
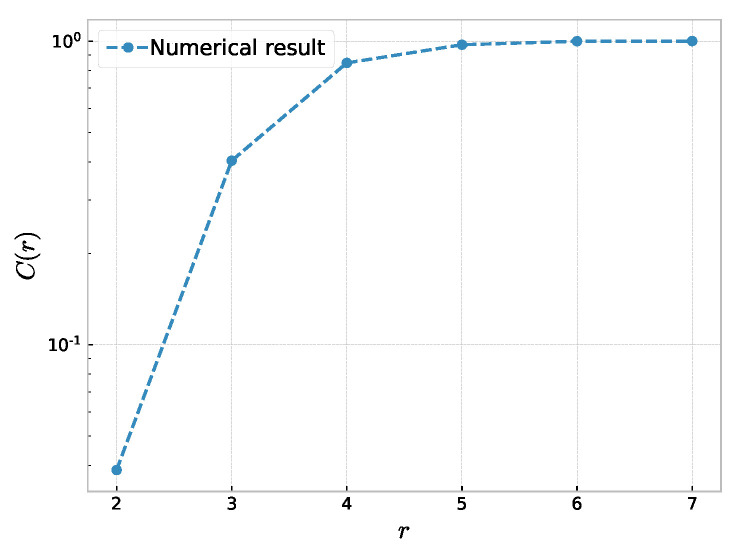
The log–log plot of scaling distance *r* and correlation sum C(r) of the USAir without considering edge-weights, i.e., all edge-weights are set to 1 for using Wang’s correlation dimension method.

**Figure 6:**
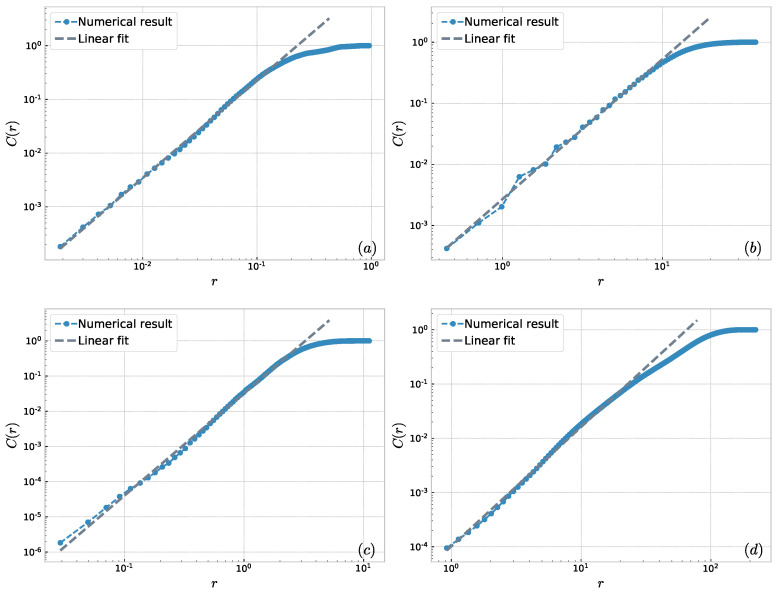
The correlation dimension of 4 real-world weighted networks: (**a**) USAir; (**b**) Netscience; (**c**) Cgscience; and (**d**) Coplant.

The USAir network has 332 nodes and 2126 edges. Furthermore, edge-weights of USAir are the number of seats available on the scheduled flights with millions per year. Then, we consider USAir as an unweighted network and calculate the values of *r* and C(r) by Wang’s method [31]. The numerical result is shown in Figure 5. An appropriate scaling region for linear fitting could not be found in the log–log plot of *r* and C(r). Wang’s method shows that the USAir network does not have fractal property. We obtained a reverse result when using our method and the result is shown in part (a) of Figure 6. It was found that USAir is a network with the fractal property. USAir has fractal properties and is also described in the references [33]. The correlation dimension of USAir is equal to 1.82, and *r* is strongly linear with C(r) on the scaling region. Therefore, the consideration of edge-weights is effective and necessary for weighted networks.

In collaboration networks, the edge-weights $w_{ij}$ represent the strength of the collaboration if any between scientists *i* and *j* is:

(7) $$w_{ij}=\sum_{k}\frac{\delta_{i}^{k}\delta_{j}^{k}}{n_{k}-1}$$

where $n_{k}$ is the number of co-authors of the paper *k*. $\delta_{i}^{k}=1$ if scientist *i* is the co-author of the paper *k*. The closer the cooperation, the larger the edge-weight. Therefore, we use the Equation (4) to obtain the shortest distance between nodes. The Netscience network has 1589 nodes and 2742 edges and Cgscience has 7343 nodes and 11,898 edges. Numerical results and fitting lines are shown in Figure 6. The results show that the two cooperative networks have fractal properties. Similarly, we found that the Coplant biological network has fractal property. We compare the information dimension and the correlation dimension of these weighted networks in Table 4. Numerical results show that the two dimensions are significantly different. The reason is that the correlation dimension and the information dimension characterize the fractal property of weighted networks from different perspectives. These dimensions can more accurately characterize the fractal and self-similarity properties of weighted complex networks from different perspectives.

## 6. Conclusions

In this paper, we extended the correlation dimension to weighted networks and discussed the factors that affect the correlation dimension of Newman–Watts small-world networks. First, we found that the increase in the correlation dimension was related to the additional edge probability. In the NW small-world, the influence of the number of neighboring nodes and network size can be quantitatively reflected by the correlation dimension and the results which are different from the volume dimension. This shows that the correlation dimension is a suitable indicator for quantitatively analyzing the small-world effects of networks. We then extend the correlation dimension to the weighted network and apply it to the analysis of two synthetic weighted fractal networks and four real-world networks. The numerical results show that the proposed method can reveal the self-similarity and fractal property of weighted networks. Meanwhile, the proposed method can also be applied to the global efficiency evaluation of complex networks, node influence identification and image processing.

## Figures and Tables

**Table 1:** The correlation dimensions of Newman–Watts small-world networks with different parameters.

*N*	*P*	k=2	k=4	k=6	k=8	k=10	k=12	k=14
1000	0	0.9928	0.9928	0.9928	0.9929	0.9929	0.9929	0.993
	0.01	1.2951	1.7943	2.3778	2.3845	2.5369	2.7175	2.8563
	0.02	2.0963	2.6473	2.7604	2.8389	3.0485	3.1493	3.2863
	0.05	2.6434	2.9127	3.0698	2.9427	3.2597	3.2817	3.6556
	0.1	2.7554	3.1952	3.2193	3.2892	3.5412	3.9118	4.1663
	0.3	3.5012	3.6739	3.8769	4.1099	4.3303	4.3404	4.3963
2000	0	0.9964	0.9964	0.9964	0.9964	0.9964	0.9964	0.9964
	0.01	2.0235	2.243	2.7706	2.9644	3.1692	3.246	3.3075
	0.02	2.2443	2.8485	2.7821	3.2911	3.2367	3.4383	3.5424
	0.05	2.8336	3.2113	3.3924	3.4125	3.5485	3.5871	3.7541
	0.1	3.3166	3.6928	3.7177	3.8836	3.8562	4.0745	4.419
	0.3	3.9042	3.8666	3.7783	4.3119	4.715	4.9166	4.9635
3000	0	0.9976	0.9976	0.9976	0.9976	0.9976	0.9976	0.9976
	0.01	2.05	2.5912	2.783	2.8573	2.9366	3.2939	3.3541
	0.02	2.6661	3.0472	3.1748	3.4464	3.5882	3.6701	3.7543
	0.05	3.2911	3.5353	3.8673	3.8855	3.9201	3.9976	4.1099
	0.1	3.6983	3.8749	3.8807	3.9527	3.9992	4.108	4.5074
	0.3	3.8757	3.9531	4.21	4.4034	4.831	5.1289	5.2853
5000	0	0.9985	0.9985	0.9985	0.9986	0.9985	0.9986	0.9986
	0.01	2.317	3.06	3.2107	3.332	3.4623	3.5619	3.6939
	0.02	2.9298	3.3299	3.6783	3.9903	4.0966	3.776	4.1588
	0.05	3.5714	3.9696	4.256	4.2406	4.3665	4.4102	4.4677
	0.1	3.9889	4.2811	4.366	4.40426	4.4924	4.502	4.5754
	0.3	4.5767	4.6517	4.8547	4.9715	5.2839	5.3029	5.5834
8000	0	0.9991	0.9991	0.9991	0.9991	0.9991	0.9991	0.9991
	0.01	2.6598	3.3849	3.5494	3.7576	3.8403	3.9471	4.1057
	0.02	3.1861	3.5808	3.9604	4.0501	4.1467	4.2819	4.3718
	0.05	3.8462	3.9158	4.1813	4.2552	4.3968	4.4726	4.5833
	0.1	4.4148	4.4527	4.5175	4.5836	4.5906	4.6994	4.8064
	0.3	4.7167	5.0379	5.0734	5.076	5.1369	5.4082	5.7126

Here, *N* represents the number of nodes, *k* represents the initial number of neighbors, *P* represents the added edge probability.

**Table 2:** The theoretical dimensions ($d_{t}$), correlation dimension ($d_{c}$), information dimension ($d_{I}$) and the dimensions calculated by BCANw method ($d_{B}$) of “Sierpinski” WFNs.

	$d_{t}$	$d_{c}$	$d_{I}$	$d_{B}$
f=1/2	1.585	1.562	1.505	1.389
f=1/3	1	0.987	0.953	0.919
f=1/4	0.792	0.822	0.803	0.776
f=1/5	0.683	0.710	0.694	0.674
f=1/6	0.613	0.613	0.590	0.579
f=1/7	0.565	0.565	0.571	0.563
f=1/8	0.528	0.529	0.530	0.526
f=1/9	0.5	0.5	0.485	0.479

**Table 3:** The theoretical dimensions ($d_{t}$), correlation dimension ($d_{c}$), information dimension ($d_{I}$) and the dimensions calculated by BCANw method ($d_{B}$) of “Cantor Dust” WFNs.

	$d_{t}$	$d_{c}$	$d_{I}$	$d_{B}$
f=1/2	2	1.863	1.857	1.704
f=1/3	1.262	1.245	1.228	1.195
f=1/4	1	1.008	1.006	0.974
f=1/5	0.861	0.873	0.871	0.849
f=1/6	0.774	0.780	0.786	0.767
f=1/7	0.712	0.719	0.725	0.771
f=1/8	0.667	0.678	0.676	0.728
f=1/9	0.631	0.637	0.642	0.685

**Table 4:** Correlation dimension ($d_{c}$) and information dimension ($d_{I}$) of real-world weighted networks. The information dimension does not exist in biological network Coplant.

Name of Network	Nodes	Edges	$d_{c}$	$d_{I}$
USAir	332	2126	1.82	1.302
Netscience	1589	2742	2.288	0.634
Cgscience	7343	11898	2.908	2.419
Coplant	2210	12188	2.199	-
